# TRPA1 as a potential factor and drug target in scleroderma: dermal fibrosis and alternative macrophage activation are attenuated in TRPA1-deficient mice in bleomycin-induced experimental model of scleroderma

**DOI:** 10.1186/s13075-023-02994-z

**Published:** 2023-01-25

**Authors:** Ilari Mäki-Opas, Mari Hämäläinen, Eeva Moilanen, Morena Scotece

**Affiliations:** 1 The Immunopharmacology Research Group, Faculty of Medicine and Health Technology, Tampere University and Tampere University Hospital, 33014 Tampere, Finland; 2 Current affiliation: Molecular Mechanisms of Cancer Program, Centro de Investigación del Cáncer (CIC), Instituto de Biología Molecular y Celular del Cáncer (IBMCC), CSIC-USAL, 37007 Salamanca, Spain

**Keywords:** Scleroderma, Systemic, Autoimmune diseases, Inflammation, TRPA1, Transient receptor potential channels, Macrophages

## Abstract

**Background:**

Systemic sclerosis is a rheumatoid disease best known for its fibrotic skin manifestations called scleroderma. Alternatively activated (M2-type) macrophages are normally involved in the resolution of inflammation and wound healing but also in fibrosing diseases such as scleroderma. TRPA1 is a non-selective cation channel, activation of which causes pain and neurogenic inflammation. In the present study, we investigated the role of TRPA1 in bleomycin-induced skin fibrosis mimicking scleroderma.

**Methods:**

Wild type and TRPA1-deficient mice were challenged with intradermal bleomycin injections to induce a scleroderma-mimicking disease. Macrophages were investigated in vitro to evaluate the underlying mechanisms.

**Results:**

Bleomycin induced dermal thickening and collagen accumulation in wild type mice and that was significantly attenuated in TRPA1-deficient animals. Accordingly, the expression of collagens 1A1, 1A2, and 3A1 as well as pro-fibrotic factors TGF-beta, CTGF, fibronectin-1 and YKL-40, and M2 macrophage markers Arg1 and MRC1 were lower in TRPA1-deficient than wild type mice. Furthermore, bleomycin was discovered to significantly enhance M2-marker expression particularly in the presence of IL-4 in wild type macrophages in vitro, but not in macrophages harvested from TRPA1-deficient mice. IL-4-induced PPARγ-expression in macrophages was increased by bleomycin, providing a possible mechanism behind the phenomenon.

**Conclusions:**

In conclusion, the results indicate that interfering TRPA1 attenuates fibrotic and inflammatory responses in bleomycin-induced scleroderma. Therefore, TRPA1-blocking treatment could potentially alleviate M2 macrophage driven diseases like systemic sclerosis and scleroderma.

## Background

Systemic sclerosis, also known as scleroderma, is a rheumatoid disease affecting about 1–4 in 10,000 people (prevalence) [1]. In the pathophysiology of the disease, fibrosis follows autoimmunity, inflammation, and vasculopathy of small arterioles. Clinical manifestations are versatile. Typical first manifestations include common symptoms such as Raynaud’s phenomenon and gastroesophageal reflux [2]. Therefore, the disease is not easy to detect early. This is one of the main challenges in the treatment of the disease, as reversible inflammation at the beginning is later followed by irreversible fibrosis of skin, namely the scleroderma, and more fatally, fibrosis of many internal organs. One of the most widely used experimental models of the disease is bleomycin-induced scleroderma, where subcutaneous injections of bleomycin induce scleroderma-like histopathologic changes imitating inflammatory and fibrotic features of the disease [3].

Macrophages are leukocytes that have versatile roles in inflammation, tissue repair, and metabolic homeostasis [4]. Inflammatory features of systemic sclerosis include overactivation of alternatively activated (M2 type) macrophages [3]. M2 macrophages are activated most famously by type 2 immunity-associated cytokines, particularly IL-4 and IL-13. M2 macrophages are referred as anti-inflammatory macrophages as they have a role in the suppression and resolution of inflammation and in the scar formation, but their overactivation may lead to unwanted fibrosis [5].

Transient receptor potential ankyrin 1 (TRPA1) is a non-selective cation channel investigated predominantly in nociceptors, but it is currently known to be expressed in some other cell types as well [6]. Non-neuronal cells expressing TRPA1 include fibroblasts [7], keratinocytes, melanocytes, and macrophages [8–11]. Although some level of activation by thermal and mechanical stimulus has been shown, TRPA1 is mainly activated by exogenous irritating substances like AITC from mustard oil and by endogenous inflammatory mediators like reactive oxygen and nitrogen species and bradykinin [12]. Opening of TRPA1 leads to an increase in the intracellular calcium which in neurons can trigger action potential and release of mediators of neurogenic inflammation such as substance P and calcitonin gene related peptide (GCRP) [13].

In the present study, we hypothesized that TRPA1 activation may have a role in macrophage polarization towards alternatively activated M2 phenotype. The hypothesis was inspired by animal studies where genetic ablation of TRPA1 was shown to exacerbate M1-driven diseases atherosclerosis and renal ischemia-reperfusion injury [14, 15]. In the earlier atherosclerosis mouse model study, macrophages from TRPA1-deficient mice were also shown to have downregulated expression of M2 markers. The present study approaches the hypothesis from a more direct angle. If TPRA1 mediates M2-macrophage activation, its inhibition should attenuate M2 driven diseases. Scleroderma is an example of such disease. Therefore, we set out to compare mice with functional and non-functional TRPA1-gene in bleomycin-induced model of scleroderma and complemented the study with in vitro experiments in cultured mouse macrophages.

## Methods

### Animals

Wild type (WT) and TRPA1-deficient male B6;129P-Trpa1(tm1Kykw)/J mice (Charles River Laboratories, Sulzfeld, Germany) were used in the bleomycin-induced experimental model of scleroderma. Mice were bred at Tampere University preclinical facility under standard conditions (12:12 light to dark cycle, 22 ± 1°C temperature, 50–60% humidity), and food and water were provided ad libitum. Animal experiments were carried out in accordance with the EU legislation for the protection of animals used for scientific purposes (Directive 2010/63/EU), and the study was approved by the National Animal Experiment Board (ESAVI/24887/2020).

### Bleomycin administration

Bleomycin (Cayman Chemical, Michigan, USA) was dissolved in sterile phosphate-buffered saline (PBS) in the final concentration of 0.5 mg/ml. The upper dorsa of mice were shaved, and a square (about 1.5 cm^2^) was drawn with a marker. Mice were anesthetized with sevoflurane inhalation, and using a 27-gauge needle, 100 ml of bleomycin was administrated i.d. in the shaved area every other day for 28 days. The bleomycin injection sites were rotated. The day after the last injection, the mice were euthanized and the injected skin was cut with a 6 mm punch. Two 6-mm skin specimens were fixed in 10% formalin and used for histological analyses. One specimen was stored in RNA-later solution (Invitrogen, Life technologies, Carlsbad, CA, USA) and processed for RNA extraction. Skin samples were obtained from TRPA1-deficient and wild type mice injected with bleomycin (*n* = 6). Control skin was collected from TRPA1-deficient and wild type mice (*n* = 6) that did not receive bleomycin treatment.

### Histological analysis

Skin samples were fixed in 10% formalin and embedded in paraffin. Sections of skin (6 mm thick) were cut, mounted on slide, and stained with hematoxylin and eosin (HE, Histolab Products AB, Göteborg, Sweden) or with Masson’s trichrome (Sigma-Aldrich Chemical Company, St. Louis, MO, USA). Dermal thickness (mm) was assessed in HE stained sections by measuring the distance between the epidermal-dermal junction and the dermal-fat junction at six randomly selected locations in each section using ImageJ software. Collagen-content was measured in Masson’s-stained sections by ImageJ software as previously described [16].

### Cell culture

Mouse J774 macrophages (American Type Culture Collection, Manassas, VA) were cultured in Dulbecco’s modified Eagle’s medium with Ultraglutamine 1 (Sigma-Aldrich, St. Louis, MO) supplemented with 10% heat-inactivated fetal bovine serum (R&D Systems Europe Ltd, Abingdon, UK), 100 U/ml penicillin, 100 μg/ml streptomycin, and 250 ng/ml amphotericin (all three from Invitrogen, Carlsbad, CA) at 37 °C in a 5% CO_2_ atmosphere. For RNA extraction and preparation of whole-cell lysates for Western blotting, the cells were cultured in 24-well plates with medium containing the compounds of interest.

Isolation and culturing of mouse peritoneal macrophages from TRPA1-deficient and wild type mice were carried out as described in Korhonen et al. 2015 [17]. In each experiment, cells from six wild type and six knock-out mice were pooled to give *n* = 4.

### RNA extraction and quantitative RT-PCR

RNA from skin samples, and from J774 and peritoneal macrophages was extracted using the GenElute Mammalian Total RNA Miniprep Kit (Sigma-Aldrich Chemical Company, St. Louis, MO, USA), according to the manufacturer’s instructions. Total RNA from skin samples and peritoneal macrophages was reverse-transcribed to cDNA by using Maxima First Strand cDNA Synthesis Kit for RT-qPCR (Thermo Fisher Scientific, Waltham, MA, USA) and from J774 macrophages by using TaqMan Reverse Transcription reagents (Applied Biosystems, Foster City, CA, USA). After the transcription reaction, the cDNA obtained was subjected to PCR using TaqMan Universal PCR Master Mix and ABI PRISM 7500 Sequence detection system (Applied Biosystems). In the case of IL-6, Arg1, COX-2, and GAPDH, the primer and probe sequences and concentrations were optimized according to the manufacturer’s guidelines in TaqMan Universal PCR Master Mix Protocol part number 4304449 revision C and are summarized in Table 1. Primers and probes were purchased from Metabion (Martinsried, Germany).

**Table 1:** Primer and probe sequences of GAPDH, IL6, Arg1, and COX-2

Primer/probe	Sequence
**Mouse glyceraldehyde-3-phosphate dehydrogenase (GAPDH)**	
Forward primer	5′-GCATGGCCTTCCGTGTTC-3′
Reverse primer	5′-GATGTCATCATACTTGGCAGGTTT-3′
Probe	5′-TCGTGGATCTGACGTGCCGCC-3′
**Mouse interleukin 6 (IL-6)**	
Forward primer	5′-TCGGAGGCTTAATTACACATGTTC-3′
Reverse primer	5′-CAAGTGCATCATCGTTGTTCATAC-3′
Probe	5′-CAGAATTGCCATTGCACAACTCTTTTCTCA-3′
**Mouse arginase 1 (Arg1)**	
Forward primer	5′-TCCAAGCCAAAGTCCTTAGAGATTAT-3′
Reverse primer	5′-CGTCATACTCTGTTTCTTTAAGTTTTTCC-3′
Probe	5′-CGCCTTTCTCAAAAGGACAGCCTCGA-3′
**Mouse cyclooxygenase 2 (COX-2)**	
Forward primer	5′-GCCAGGGCTGAACTTCGAA-3′
Reverse primer	5′-CAATGGGCTGGAAGACATATCAA-3′
Probe	5′-CTCACGAGGCCACTGATACCTATTGCATTG-3′

TaqMan Gene Expression assays for mouse collagen type I alpha 1 chain (COL1A1) (Mm00801666_g1), mouse collagen type I alpha 2 chain (COL1A2) (Mm00483888_m1), mouse collagen type III alpha 1 chain (COL3A1) (Mm01254476_m1), mouse transforming growth factor beta 1 (TGF-b1) (Mm01178820_m1), mouse fibronectin-1 (Mm01256744_m1), mouse connective tissue growth factor (CTGF) (Mm01192933_g1), mouse chitinase-3-like protein 1 (CHI3L1) also known as YKL-40 (Mm00801477_m1), mouse interleukin-13 (IL-13) (Mm00434204_m1), mouse mannose receptor C-type 1 (MRC-1) (Mm01329362_m1), mouse peroxisome proliferator-activated receptor gamma (PPARγ) (Mm01184322_m1), and mouse monocyte chemoattractant protein 1 **(**MCP-1) (Mm00441242_m1) were obtained from ThermoFisher Scientific, Waltham, MA, USA. The PCR cycling parameters were as follows: incubation at 50 °C for 2 min, incubation at 95 °C for 10 min, and thereafter 40 cycles of denaturation at 95 °C for 15 s and annealing and extension at 60 °C for 1 min. The mRNA levels were normalized against the housekeeping gene GAPDH mRNA levels and quantified using the ∆∆Ct method.

### Protein extraction and Western blotting

Cells were incubated in the presence or absence of IL-4 (1 ng/ml) and treated with bleomycin (10 μg/ml) for the indicated time. Thereafter, whole-cell lysates were prepared and Western blot carried out as previously described [18] with primary antibodies #56554 for phospho-STAT6 and #9362 for STAT6 from Cell Signaling Technology (Beverly, MA, USA).

### Statistical analysis

The results are presented as the mean + standard error of the mean (SEM). One or two way analysis of variance (ANOVA) followed by Tukey’s or Bonferroni’s multiple comparison test was used. *p* values less than 0.05 were considered significant. Data were analyzed using the Prism computerized package (GraphPad Software, San Diego, CA, USA).

## Results

### TRPA1-deficient mice have attenuated bleomycin-induced dermal thickening

**Fig. 1:**
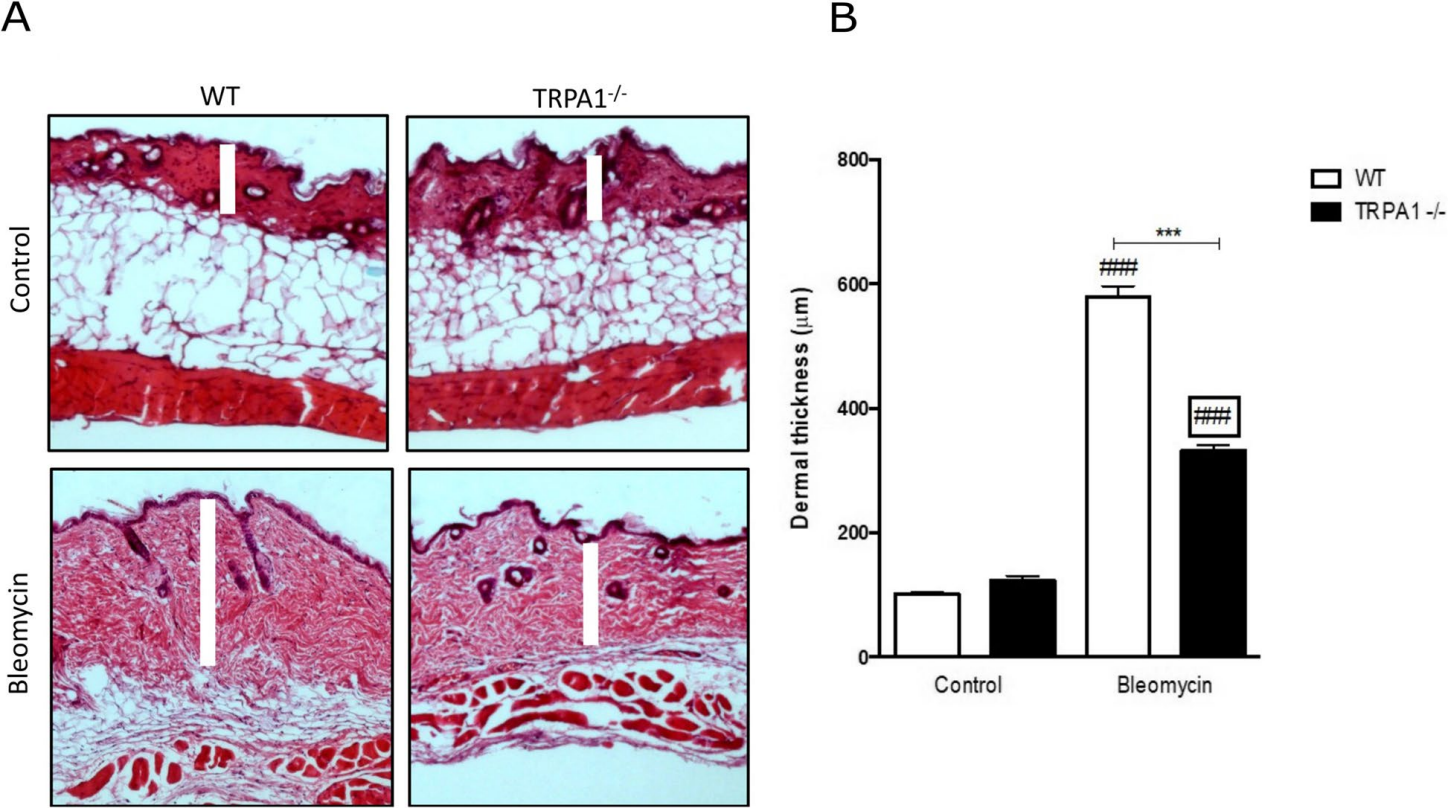
TRPA1-deficient mice presented attenuated dermal thickening in response to bleomycin injections. Skin sections of control and bleomycin treated wild type (WT) and TRPA1-deficient (TRPA1^−/−^) mice were stained with hematoxylin and eosin. Representative images are shown (**A**) and data of dermal thickness measurements are summarized as a graph (**B**). Dermal thickness (rectangular blank bar in **A**) was calculated by averaging the thickness measured at six randomly selected locations in each section using the ImageJ software. Bars represent means of the dermal thickness + SEM; *n*  =  6. ***p < 0.001 between bleomycin-treated WT and TRPA1-deficient mice; ^###^*p* < 0.001 between control and bleomycin-treated mice within the same genotype

To determine the role of TRPA1 in bleomycin-induced scleroderma, skin specimens were collected from WT and TRPA1-deficient mice and stained with hematoxylin and eosin to evaluate the dermal thickness. WT and TRPA1-deficient control mice did not present any difference in dermal thickness (Fig. 1). Interestingly, when analyzing the mice treated with bleomycin, we observed a clearly and significantly attenuated increase in the dermal thickness in TRPA1-deficient mice compared with WT mice (Fig. 1).

### Bleomycin-induced collagen accumulation is attenuated in TRPA1-deficient mice

**Fig. 2:**
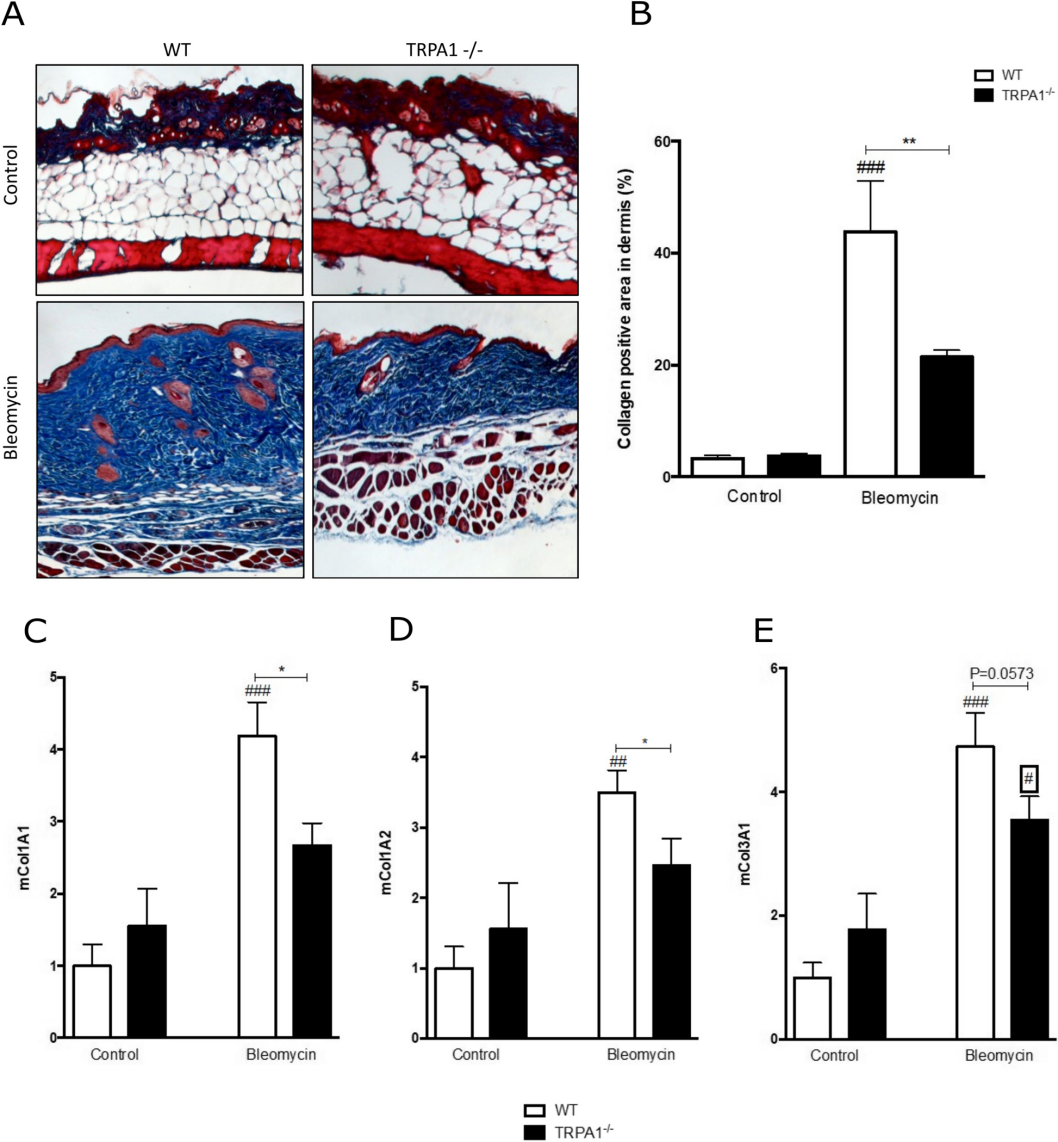
Bleomycin-induced collagen accumulation was attenuated in the skin of TRPA1-deficient mice. Representative images of Masson’s trichrome-stained sections of mouse skin harvested after 28 days of treatment with bleomycin or from control mice (**A**). Graphical representation of collagen measurement (**B**). Collagen content was measured using the ImageJ software. Col1A1 (**C**), Col1A2 (**D**), and Col3A1 (**E**) mRNA expression in the skin from WT and TRPA1-deficient (TRPA^−/−^) mice treated with bleomycin and in corresponding controls were measured by RT-PCR and normalized against GAPDH mRNA. WT control was given the value of 1 and the other values are set in the proportion to that value. Bars represent means of the collagen content + SEM; *n*  =  6. *p < 0.05, **p < 0.01 between bleomycin treated WT and TRPA1-deficient mice; ^###^*p* < 0.001 and ^#^*p* < 0.05 between control and bleomycin treated mice within the same genotype

We investigated the collagen content in the mouse skin by using Masson’s trichrome stain. As shown in the histological images (Fig. 2 A), and in the bar graph (Fig. 2 B), collagen content in the skin was increased in bleomycin treated mice as compared to the control mice (Fig. 2 A and B). Interestingly, collagen accumulation in bleomycin-treated TRPA1-deficient mice was significantly lower than in the WT counterparts. No difference was detected between WT and TRPA1-deficient control groups (Fig. 2 A and B).

Consistently with the reduced collagen content measured by histology, we also found that TRPA1-deficient mice presented a significantly lower expression of collagens Col1A1 (Fig. 2 C), Col1A2 (Fig. 2 D), and Col3A1 (Fig. 2 E) in the bleomycin-treated skin as compared with WT mice.

### Bleomycin-induced expression of pro-fibrotic mediators is lower in TRPA1-deficient mice

**Fig. 3:**
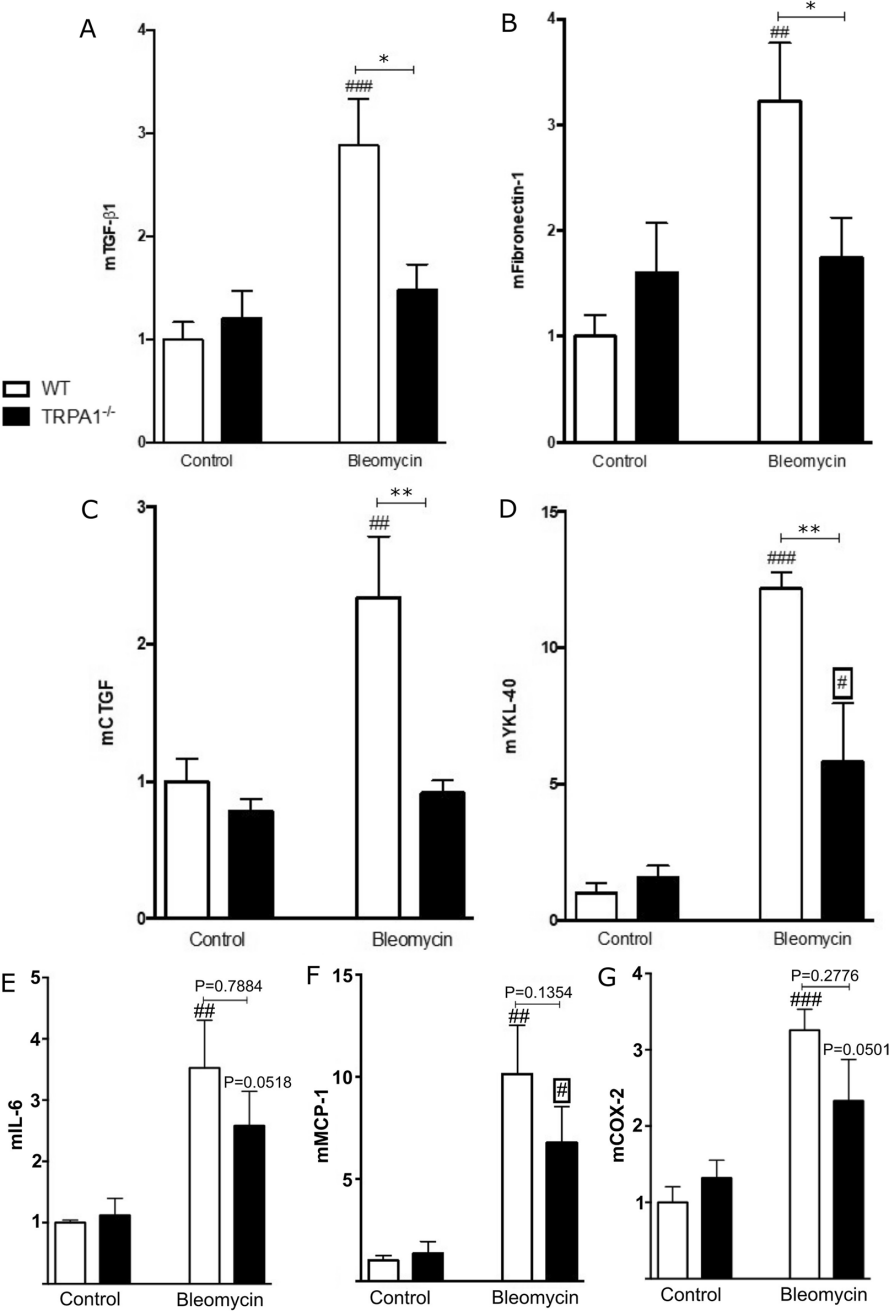
Pro-fibrotic factor expression in the skin of bleomycin-treated TRPA1-deficient mice was inhibited. TGF-β1 (**A**), fibronectin-1 (**B**), CTGF (**C**), YKL-40 (**D**), IL-6 (**E**), MCP-1 (**F**), and COX-2 (**G**) expression in the skin from WT and TRPA1-deficient (TRPA1^−/−^) mice treated with bleomycin were assessed by RT-PCR and normalized against GAPDH mRNA. WT control was given the value of 1 and the other values are set in the proportion to that value. Data are expressed as mean + SEM; *n* =  6. *p < 0.05, **p < 0.01 between bleomycin treated WT and TRPA1-deficient mice; ^#^*p* < 0.05, ^##^*p* < 0.01, ^###^*p* < 0.001, *p* = 0.0518, *p* = 0.0501 between control and bleomycin treated mice within the same genotype

To further elucidate the role of TRPA1 in bleomycin-induced skin fibrosis, we examined the expression of pro-fibrotic mediators in the skin samples. As reported in the Fig. 3, the expression of TGF-b1 (A), fibronectin-1 (B), CTGF (C), and YKL-40 (D) was significantly lower in the skin from bleomycin-treated TRPA1-deficient mice than in the samples from WT counterparts (Fig. 3). Moreover, we investigated the expression of inflammatory factors IL-6 (E), MCP-1 (F), and COX-2 (G) and observed that these mediators tended to be lower in the skin from bleomycin-treated TRPA1-deficient mice compared with bleomycin-treated WT mice, but the difference did not reach statistical significance.

### Markers of M2-type macrophage activation are lower in bleomycin-treated TRPA1-deficient mice

**Fig. 4:**
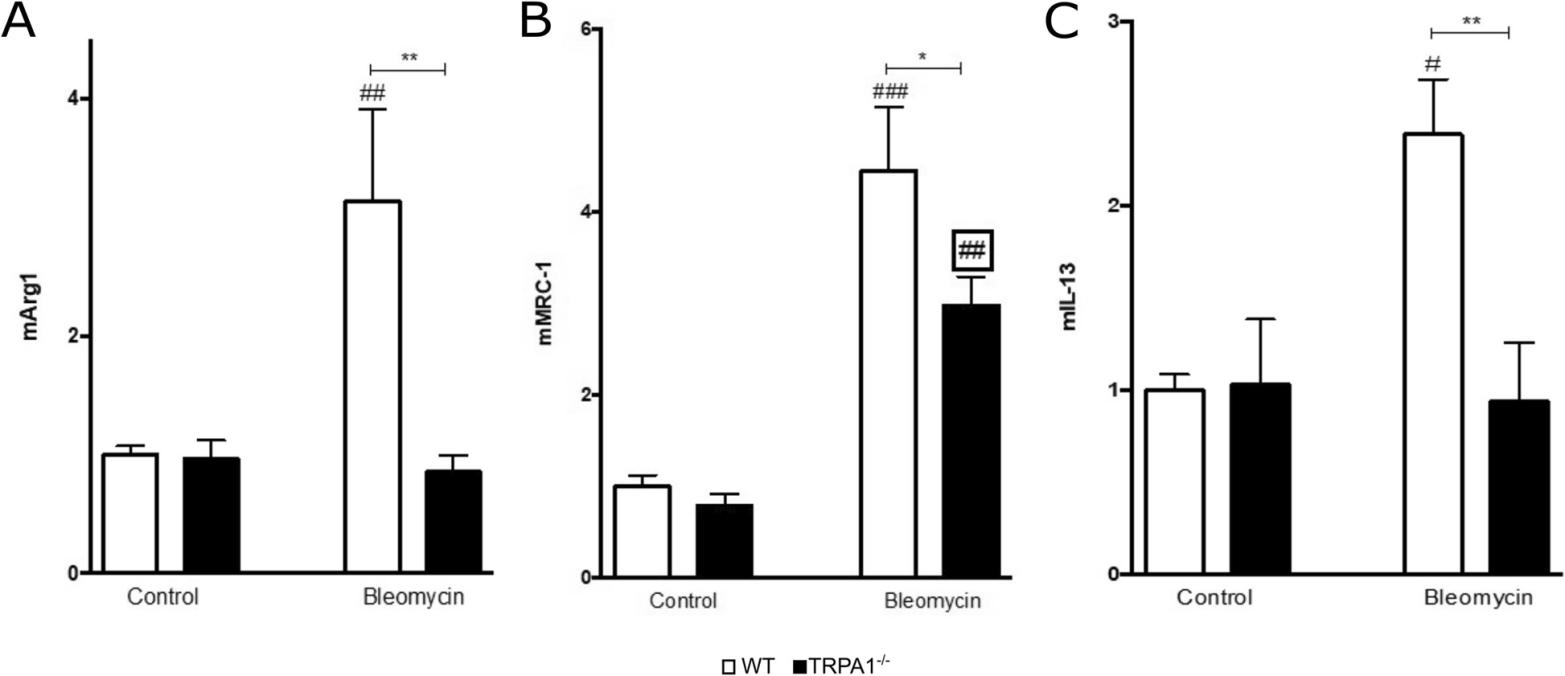
Expression of M2-type macrophage markers in response to bleomycin treatment was lower in TRPA1-deficient mice. Expression of arginase-1 (**A**), MRC-1 (**B**), and IL-13 (**C**) mRNA in the skin from WT and TRPA1-deficient (TRPA1^−/−^) mice treated with bleomycin was measured by RT-PCR. The values of the mRNA of interest were normalized against GAPDH mRNA. WT control was given the value of 1 and the other values are set in the proportion to that value. Data are expressed as mean + SEM; *n*  =  6. *p < 0.05, **p < 0.01 between bleomycin treated WT and TRPA1-deficient mice; ^#^*p* < 0.05, ^##^*p* < 0.01, ^###^*p* < 0.001 between control and bleomycin treated mice within the same genotype

Considering the critical role of alternatively activated M2-type macrophages in tissue fibrosis, we also investigated the characteristic M2 markers in the skin from WT and TRPA1-deficient mice injected with bleomycin. As reported in the Fig. 4, the expression of arginase-1 (A), MRC-1 (B), and IL-13 (C) was increased in the skin from bleomycin-treated WT mice as compared to samples from control mice (Fig. 4). Interestingly, the expression of these factors was significantly lower in the skin from bleomycin-treated TRPA1-deficient mice than in the skin samples from WT mice, indicating that TRPA1 deficiency reduces M2-macrophages contributing to its anti-fibrotic effect in bleomycin-induced dermal fibrosis.

### Bleomycin augments macrophage polarization towards M2 phenotype

**Fig. 5:**
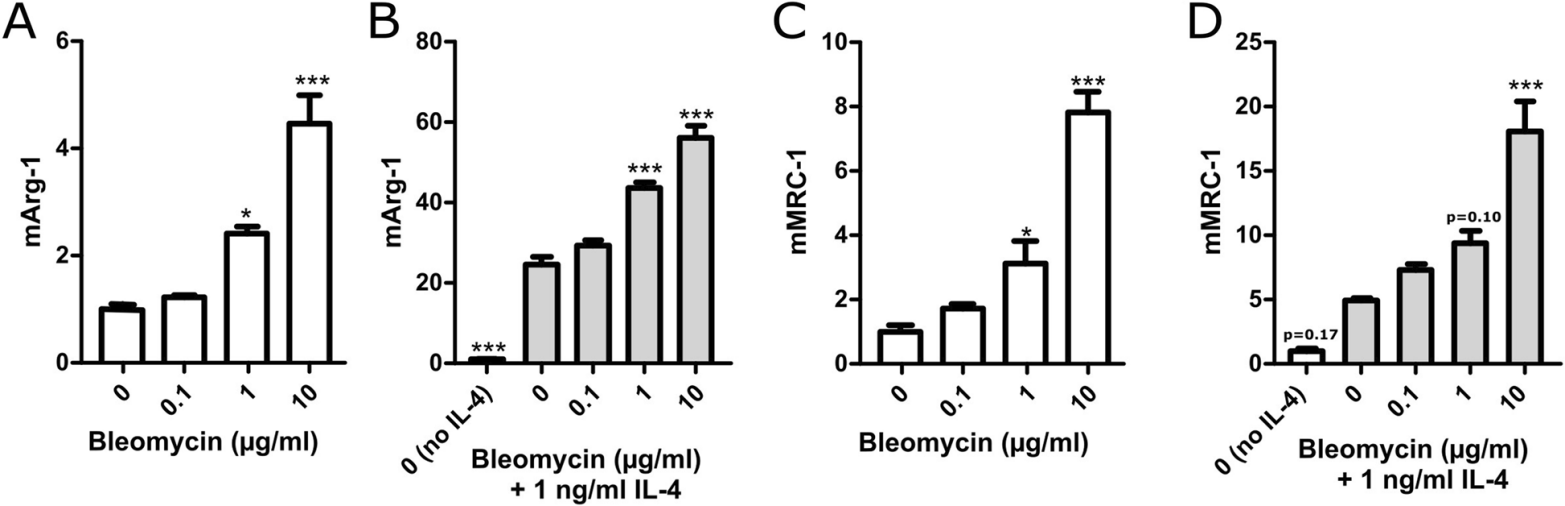
Bleomycin enhanced the expression of M2 markers in J774 macrophages with or without IL-4. J774 macrophages were incubated for 24 h with increasing concentrations of bleomycin in the absence and in the presence of IL-4 (1 ng/ml). Arginase-1 (mArg-1; **A** and **B**) and mannose receptor C type 1 (mMRC-1; **C** and **D**). mRNA levels were measured with RT-PCR and normalized to GAPDH mRNA. WT control was given the value of 1 and the other values are set in the proportion to that value. Results are presented as mean + SEM; *n* = 4. *p < 0.05, **p < 0.01, ***p < 0.001 between the control (0) and the value beneath

We set up an in vitro protocol to investigate the effect of bleomycin on M2-type macrophage activation. J774 mouse macrophages were incubated with bleomycin for 24 h before the expression of M2 markers was measured using RT-PCR. Bleomycin enhanced the expression of arginase-1 and MRC-1 in a dose-dependent manner in macrophages in the presence of a low concentration of the known M2-inducing cytokine IL-4 (Fig. 5 A and D). In the absence of IL-4, bleomycin had a minor yet statistically significant M2-polarizing effect at 1–10 μg/ml concentrations (Fig. 5 A and C).

**Fig. 6:**
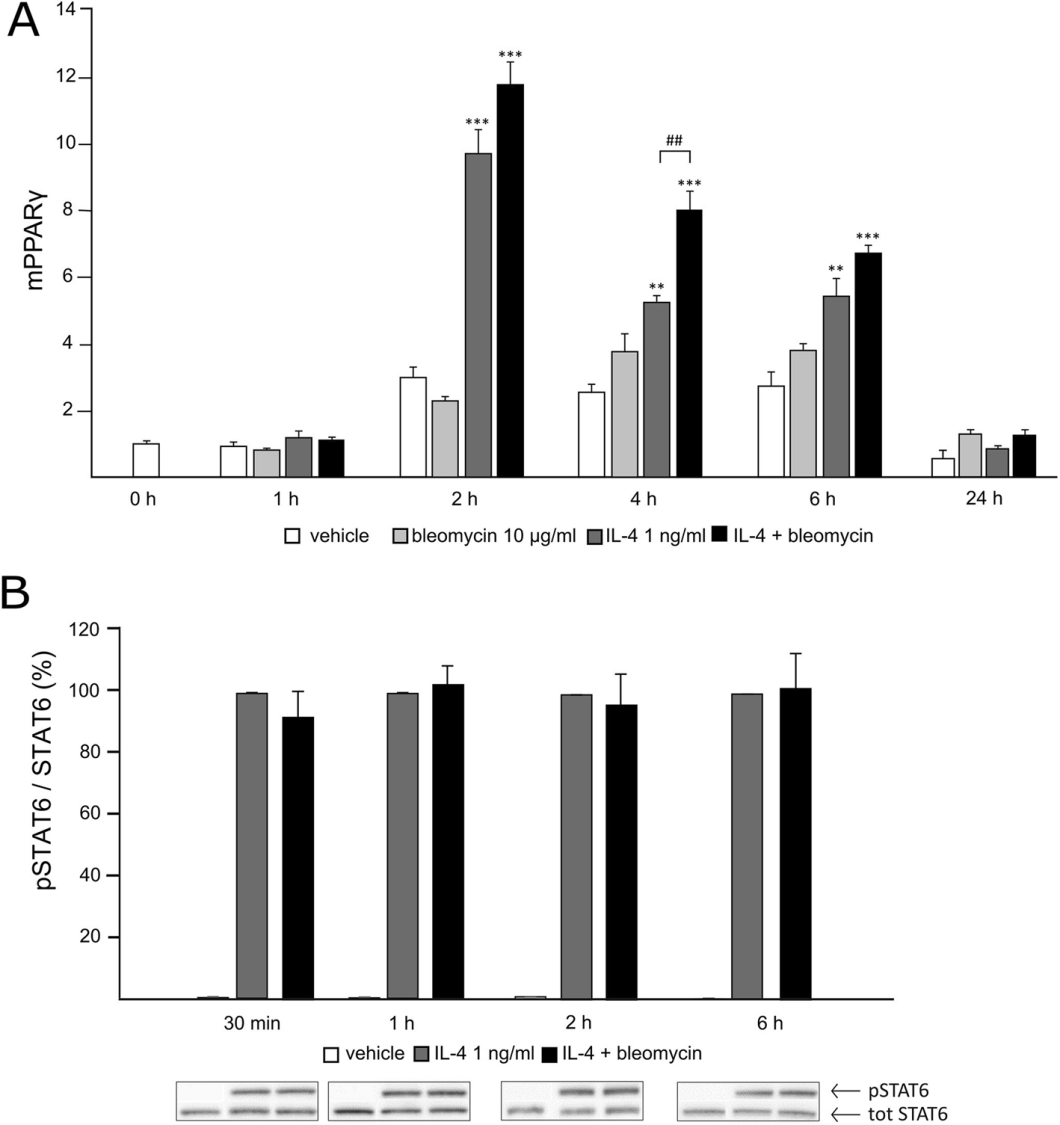
IL-4-induced expression of PPARγ was enhanced by bleomycin; no effect on STAT6 phosphorylation. J774 cells were incubated with bleomycin and/or IL-4 for the indicated time. In **A**: PPARγ mRNA levels were measured with RT-PCR and normalized to GAPDH mRNA. Untreated control cells were given the value of 1 and other values are set in the proportion to that value. Data are expressed as mean + SEM, *n* = 4. *p < 0.05, **p < 0.01, ***p < 0.001 vs. untreated control cells (vehicle group) in the same time point. ^##^*p* < 0.01 between the marked pair of values (**A**). In **B**: pSTAT6 protein levels were assessed using Western blot and normalized to total STAT6. pSTAT6/total STAT6 ratio in the IL-4-treated cells at each timepoint was set as 100% and the other values are presented in relation to that value. Bleomycin had no statistically significant effects when added on top of IL-4

To further investigate the mechanisms of the bleomycin-amplified M2-type activation, we measured the effects of bleomycin on two essential intracellular signaling mechanisms involved in the process, namely STAT6 and PPARγ. PPARγ expression was enhanced by IL-4 as expected. Interestingly, bleomycin further enhanced IL-4-induced PPARγ expression, and the effect was statistically significant when measured after 4 h incubation (Fig. 6 A). Bleomycin tended to increase PPARγ expression also in the absence of IL-4 but the effect did not reach statistical significance.

STAT6 phosphorylation was measured using Western blot. IL-4 induced a significant increase in STAT6 phosphorylation, but bleomycin had no effect in the presence or absence of IL-4 (Fig. 6 B).

### Genetic deletion of TRPA1 reduced IL-4 and bleomycin induced expression of M2-markers arginase-1 and MRC-1

**Fig. 7:**
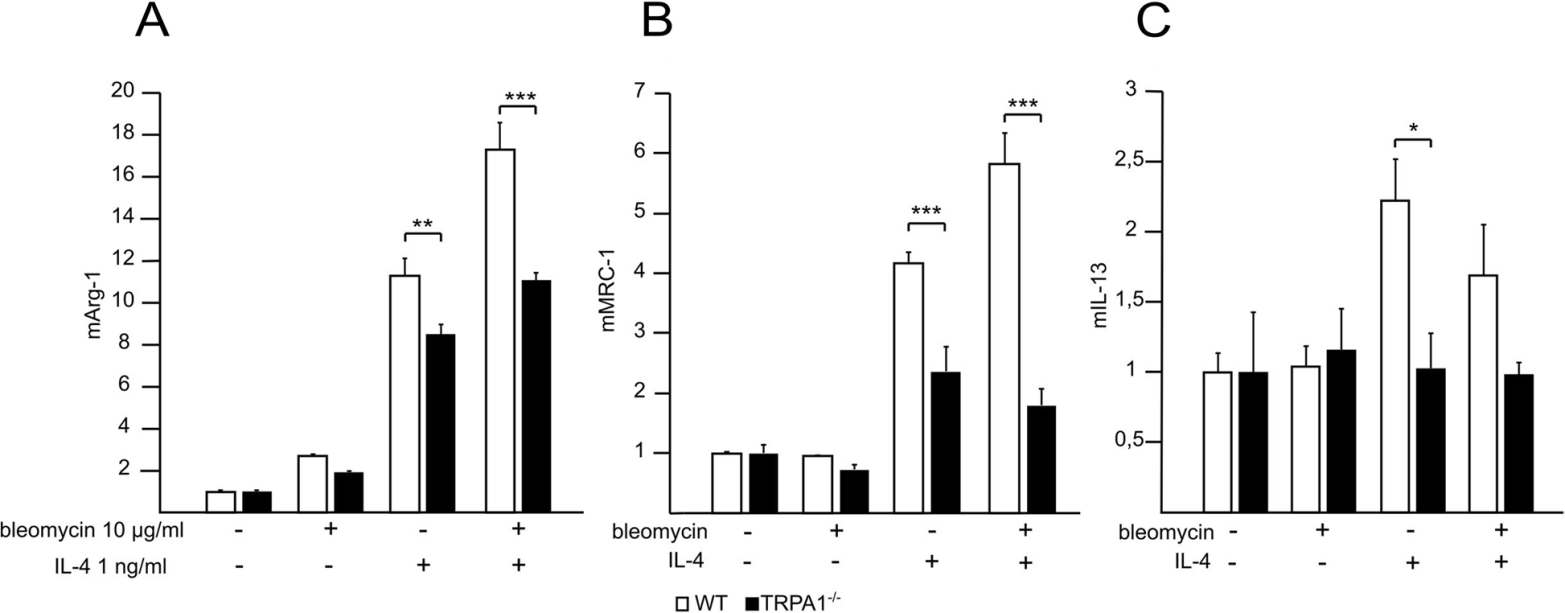
Expression of M2-markers in response to IL-4/bleomycin treatment was lower in macrophages from TRPA1-deficient mice. Mouse peritoneal macrophages were incubated with bleomycin, IL-4, or their combination for 24 h. mRNA levels were measured with RT-PCR and normalized to GAPDH mRNA. WT control was given the value of 1 and other values are set in the proportion to that value. Data are expressed as mean + SEM. *p < 0.05, **p < 0.01, ***p < 0.001

Peritoneal macrophages from WT and TRPA1-deficient mice were incubated for 24 h with bleomycin, IL-4, or their combination. Similarly with the results in the J774 macrophage experiments, M2-markers arginase-1 and MRC-1 were upregulated by IL-4 in macrophages from WT mice, and this effect was enhanced by bleomycin. Interestingly, in primary peritoneal macrophages from TRPA1-deficient mice, the expression of the M2 markers was significantly less enhanced in response to IL-4 and IL-4 + bleomycin treatment (Fig. 7 A and B).

IL-4 stimulated also IL-13 expression in WT peritoneal macrophages, but this effect was not seen in the TRPA1-deficient cells, suggesting a TRPA1-mediated mechanism (Fig. 7 C).

## Discussion

In the present study, we show first evidence of the involvement of TRPA1 in bleomycin-induced model of scleroderma. In histological analysis of bleomycin-challenged mouse skin, there was a significant difference in the skin thickness and collagen accumulation when comparing wild type and TRPA1-deficient mice. Attenuated collagen expression in the skin samples from TRPA1-deficient mice was also seen in RT-PCR measurements. These findings indicate that the presence of TRPA1 amplifies histologic manifestations that are presented in scleroderma. Collagen accumulation is a main characteristic of scleroderma. In patients, it is most prominent in reticular dermis and adipose tissue underneath is often involved as collagen entraps adipose cells [3]. This can be also seen in our histologic preparations of mouse skin and those changes were less severe in TRPA1-deficient mice.

We also investigated the expression of genes related to fibrosis and M2-type macrophage activation in bleomycin-challenged mouse skin. Bleomycin exacerbated the expression of pro-fibrotic factors transforming growth factor beta (TGF-β), fibronectin 1, and connective tissue growth factor (CTGF) in wild type mice skin samples, but this was not seen in samples from TRPA1-deficient mice, which suggests a shutdown of those pathways. YKL-*40* mRNA levels were significantly upregulated in both genotypes, but this phenomenon was more modest in TRPA1-deficient mice. TGF-β, fibronectin 1, and CTGF are pro-fibrotic factors that cause loss of adipogenic dermal fibroblasts [19], catalyze collagen binding [20], and enhance extracellular matrix production [21] respectively. YKL-40 has been proposed to be a driver of Th2 inflammation and M2-type macrophage activation in mouse skin inflammation [22].

Alternatively activated (M2-type) macrophages are major facilitators of wound healing and fibrosis [4, 23], and therefore, we investigated the presence of M2-activation markers in the bleomycin-challenged skin. Two of the most widely used mouse M2-markers arginase 1 and mannose receptor C type 1 (MRC-1) were measured. Bleomycin treatment enhanced the expression of these M2 activation markers in skin samples from wild type mice, but the response was significantly less severe in the samples from TRPA1-deficient mice. Arginase 1 upregulation by bleomycin was not even noticeable in knock-out mice. Arginase 1 is secreted by M2-type macrophages in mice and promotes fibrosis via its secondary metabolite ornithine that boosts collagen biosynthesis leading to fibrosis [24]. Similarly attenuated was IL-13 expression, which is an interleukin released by Th2-cells and is one of the main inducers of M2 activation [3, 25]. These results suggest that TRPA1 activation supports M2-polarization of macrophages.

The three main components of this study’s hypothesis are bleomycin, macrophages and TRPA1. In vitro methods were used here to focus on these specific factors. In experiments carried out by using J774 murine macrophages, it was found that bleomycin, though not solely a strong inducer of M2-markers, amplifies IL-4-induced M2-type macrophage activation. The interaction between IL-4 and bleomycin was statistically significant when tested using two-way analysis of variance. This effect has not been described previously and extends our understanding on the mechanisms of bleomycin-induced scleroderma. In a smaller scale, also bleomycin alone had a statistically significant effect upregulating M2-markers in J774-macrophages.

STAT6 and PPARγ are two major intracellular signaling pathways supporting M2-type macrophage polarization [26]. STAT6 was ruled out as a likely mechanism behind the effect of bleomycin, whereas PPARγ was identified as a potential mediator. We found that 2 h after challenge with a combination of IL-4 and bleomycin PPARγ mRNA levels were higher than when treated with IL-4 alone. The difference was the most significant at the 4 h timepoint. Prolonging the action of PPARγ and increasing its total effect is therefore suggested to be involved in the potential mechanisms by which bleomycin induces M2-type activation of macrophages.

To investigate effects of TRPA1 deficiency in in vitro models on IL-4 + bleomycin challenged macrophages, primary macrophages collected from peritoneal cavity of WT and TRPA1-deficient mice were utilized. In primary macrophages of wild type mice, the same phenomenon of bleomycin boosting the effect of IL-4 was repeated. In macrophages of TRPA1-deficient mice, however, the interaction was missing. Therefore, we conclude that bleomycin interacts with IL-4 in a TRPA1-mediated mechanism.

TRPA1 has been shown to be involved in inflammation, especially in the skin [27]. Many inflammatory modulators, most famously bradykinin, sensitize TRPA1 [12, 28]. The release of mediators of neurogenic inflammation, such as substance P and calcitonin gene related peptide, from nerve endings due to TRPA1 activation is accompanied with modulation of many genes affecting cutaneous inflammation [27]. In addition to cutaneous nerve endings, human melanocytes [9, 29] and keratinocytes [8, 9] also express TRPA1 [9]. There are only a few studies investigating the action of TRPA1 on monocytes and macrophages: LPS-induced nuclear factor κ B-mediated promoter activity and expression of inducible nitric oxide synthase, COX-2, and tumor necrosis factor alpha are downregulated by TRPA1 agonist cinnamaldehyde [30]. This has been interpreted as anti-inflammatory action, but it also fits well to the hypothesis that TRPA1 activation drives towards M2 macrophage polarity [27, 31].

A body of research supports the important role of macrophages in inflammation and fibrosis in systemic sclerosis. It has been shown that skin of patients suffering from systemic sclerosis has increased numbers of macrophages. The patients also have higher levels of CD163, an M2 marker, in their serum and skin [32, 33]. Nintedanib is a drug used to treat systemic sclerosis associated fibrosis and has been shown to downregulate M2 macrophage activation in mice [34, 35].

In the present study, we focused mainly on M2-type macrophages as potential targets of TRPA1 in bleomycin-induced model of fibrosis. Weaknesses of our study include, however, missing macrophage marker stained skin samples using combinations of M1 and M2 specific surface protein antibodies [36]. Those experiments should be included in future studies to confirm the present results. Our model has features of an IL-4 mediated fibrosis which mainly results as collagen accumulation, but TGF-β mediated fibrosis could also be studied in greater detail including also additional markers such as α-smooth muscle actin [35, 37]. In addition, as TRPA1 is highly permeable to calcium and expressed in fibroblasts, it is an interesting hypothesis for future studies if TRPA1-antagonists could alleviate fibrosis by influencing calcium signaling of fibroblasts like some other compounds have been reported to do [38, 39]. Intracellular calcium mediated fibrosis induced by activation of TRPA1 in fibroblasts has also been reviewed recently [7].

## Conclusions

The present results extend the previous understanding by showing that TRPA1 is involved in the development of bleomycin-induced skin fibrosis. We propose that TRPA1 influences inflammatory responses in bleomycin-induced scleroderma by boosting activation of M2-type macrophages and other cell types including fibroblasts leading to fibrosis; therefore, TRPA1 antagonists may have therapeutic potential in fibrosing diseases.

## Data Availability

The datasets supporting the conclusions of this study are included within the article.

## Abbreviations

ANOVA: Analysis of variance
Arg1: Arginase 1
CGRP: Calcitonin gene-related peptide
COL1A1: Collagen type I alpha 1 chain
COL1A2: Collagen type I alpha 2 chain
COL3A1: Collagen type III alpha 1 chain
COX-2: Cyclooxygenase 2
CTGF: Connective tissue growth factor
GAPDH: Glyceraldehyde-3-phosphate dehydrogenase
IL-13: Interleukin 13
IL-6: Interleukin 6
M2-type macrophages: Alternatively activated macrophages
MCP-1: Monocyte chemoattractant protein 1
MRC1: Mannose receptor C-type 1
PPARγ: Peroxisome proliferator-activated receptor gamma
SEM: Standard error of the mean
TGF-β1: Transforming growth factor beta 1
TRPA1: Transient receptor potential ankyrin 1
YKL-40/CHI3L1: Chitinase-3-like protein 1.
